# Supplementary material for: Inflammatory and neurodegenerative serum protein biomarkers increase sensitivity to detect clinical and radiographic disease activity in multiple sclerosis
Source: Nat Commun. 2024 May 20;15:4297. doi: 10.1038/s41467-024-48602-9 (PMC11106245; doi:10.1038/s41467-024-48602-9)
Supplement: Supplementary file 2 — Reporting Summary [file 41467_2024_48602_MOESM2_ESM.pdf]

nature portfolio

Corresponding author(s): Ferhan Qureshi

Last updated by author(s): 3/20/2024

Reporting Summary

Nature Portfolio wishes to improve the reproducibility of the work that we publish. This form provides structure for consistency and transparency in reporting. For further information on Nature Portfolio policies, see our Editorial Policies and the Editorial Policy Checklist.

Statistics

For all statistical analyses, confirm that the following items are present in the figure legend, table legend, main text, or Methods section.

n/a	Confirmed	
☐	☒	The exact sample size (*n*) for each experimental group/condition, given as a discrete number and unit of measurement
☐	☒	A statement on whether measurements were taken from distinct samples or whether the same sample was measured repeatedly
☐	☒	The statistical test(s) used AND whether they are one- or two-sided<br>*Only common tests should be described solely by name; describe more complex techniques in the Methods section.*
☐	☒	A description of all covariates tested
☐	☒	A description of any assumptions or corrections, such as tests of normality and adjustment for multiple comparisons
☐	☒	A full description of the statistical parameters including central tendency (e.g. means) or other basic estimates (e.g. regression coefficient) AND variation (e.g. standard deviation) or associated estimates of uncertainty (e.g. confidence intervals)
☐	☒	For null hypothesis testing, the test statistic (e.g. *F*, *t*, *r*) with confidence intervals, effect sizes, degrees of freedom and *P* value noted<br>*Give P values as exact values whenever suitable.*
☒	☐	For Bayesian analysis, information on the choice of priors and Markov chain Monte Carlo settings
☐	☒	For hierarchical and complex designs, identification of the appropriate level for tests and full reporting of outcomes
☐	☒	Estimates of effect sizes (e.g. Cohen's *d*, Pearson's *r*), indicating how they were calculated

Our web collection on statistics for biologists contains articles on many of the points above.

Software and code

Policy information about availability of computer code

Data collection	No software was used.
Data analysis	Protein analysis was completed using Proximity Extension Assay technology on the Olink™ Platform, xMAP® technology immunoassays at Myriad RBM, Inc. (RBM). All analyses were performed in the python programming language (version 3.11.7). SciPy version 1.11.4 was utilized for statistical tests and Scikit-Learn version 1.2.2 was utilized for machine learning models. Expanded graph techniques were completed using the Scalable Precision Medicine Open Knowledge Engine (SPOKE). Protein-protein interaction modeling was performed by inputting proteins into STRING for network construction. Topological surveillance and centrality metric calculations were performed in Cytoscape. Enrichr was used to functionally annotate protein subgraphs.

For manuscripts utilizing custom algorithms or software that are central to the research but not yet described in published literature, software must be made available to editors and reviewers. We strongly encourage code deposition in a community repository (e.g. GitHub). See the Nature Portfolio guidelines for submitting code & software for further information.

Data

Policy information about availability of data

All manuscripts must include a data availability statement. This statement should provide the following information, where applicable:

- Accession codes, unique identifiers, or web links for publicly available datasets
- A description of any restrictions on data availability
- For clinical datasets or third party data, please ensure that the statement adheres to our policy

Octave Bioscience, Inc will consider access to data from this study for qualified scientific researchers for the purpose of conducting legitimate scientific research. The company protects the rights and privacy of patient-level data. Requests for access to data can be made to fqureshi@octavebio.com, which will initiate the data request process. Responses to requests will be made within 30 days of receipt. Data presented in figures are available as a Source Data file.

Research involving human participants, their data, or biological material

Policy information about studies with human participants or human data. See also policy information about sex, gender (identity/presentation), and sexual orientation and race, ethnicity and racism.

Reporting on sex and gender	Descriptive statistics related to sex distribution in each of the three cohorts is recorded in Table 3. Demographic and clinical breakdown of all three patient cohorts. Protein expression values were corrected for sex (and other demographic variables) as part of the multivariate analysis for each clinical and radiographic endpoint.
Reporting on race, ethnicity, or other socially relevant groupings	No analyses of race, ethnicity, or socially relevant groups were performed
Population characteristics	This study used serum samples from three large, well-characterized cohorts of patients with MS participating in studies at other research sites. Population characteristics are detailed in updated version of Table 3 of the manuscript.
Recruitment	Serum samples were obtained from 3 deeply phenotyped retrospective (biobanked) cohorts from the following studies:<br>1. Comprehensive Longitudinal Investigation of MS at Brigham and Women's Hospital (CLIMB). CLIMB Endpoints: radiographically defined relapse status using Gd lesions (primary), ARR (secondary).<br>2. Expression, Proteomics, Imaging, Clinical at UCSF (EPIC). EPIC Endpoint: radiographically defined relapse status using Gd lesions.<br>3. Accelerated Cure Project (ACP). ACP Endpoint: Clinically Defined Relapse Status - active versus inactive.
Ethics oversight	The study protocol and study procedures were approved by institutional review boards and independent ethics committees at each study site. The University of California San Francisco Institutional Review Board granted ethical approval for the EPIC Cohort. Massachusetts General Brigham Human Research Committee granted ethical approval for the CLIMB Cohort. Western Institutional Review Board, Copernicus Group IRB, Sheperd Center Research Review Committee, Institutional Review Board for Human Research at St. Joseph's Hospital and Medical Center, University of Massachusetts Medical School Committee for the Protection of Human Subjects in Research, Ohio State University Biomedical Institutional Review Board, Beth Israel Deaconess Medical Center Committee on Clinical Investigations, John Hopkins medicine Office of Human Subjects Research Institutional Review Board, and the Southwestern Medical Center Institutional Review Board all granted ethical approval for the ACP Cohort.

Note that full information on the approval of the study protocol must also be provided in the manuscript.

Field-specific reporting

Please select the one below that is the best fit for your research. If you are not sure, read the appropriate sections before making your selection.

☒ Life sciences ☐ Behavioural & social sciences ☐ Ecological, evolutionary & environmental sciences

For a reference copy of the document with all sections, see nature.com/documents/nr-reporting-summary-flat.pdf

Life sciences study design

All studies must disclose on these points even when the disclosure is negative.

Sample size	Over 1400 serum proteins were evaluated in 630 samples from three MS cohorts to identify biomarkers of clinical and radiographic (gadolinium-enhancing lesions) new MS DA, which provided a large enough sample to develop the custom assay panel (CAP). Sample size was maximized or balanced based on the primary analyses being performed.
Data exclusions	For the CAP proteins, GFAP was only added to our panel after much of the development work described in this report was completed because of its strong association with several DA- and DP-related endpoints. Therefore, it was not part of the analysis. The remaining 20 proteins were carried forward.

Replication	The study utilized cross validation by bootstrap analysis and all validation attempts were successful .
Randomization	This was not a randomized trial. The data for each endpoint was randomly split into train (two-thirds) and test (one-third) subsets 1000 times.
Blinding	Patient samples used were anonymised, so blinding of patient information was not necessary.

Reporting for specific materials, systems and methods

We require information from authors about some types of materials, experimental systems and methods used in many studies. Here, indicate whether each material, system or method listed is relevant to your study. If you are not sure if a list item applies to your research, read the appropriate section before selecting a response.

Materials & experimental systems

n/a	Involved in the study
☐	☒ Antibodies
☒	☐ Eukaryotic cell lines
☒	☐ Palaeontology and archaeology
☒	☐ Animals and other organisms
☒	☐ Clinical data
☒	☐ Dual use research of concern
☒	☐ Plants

Methods

n/a	Involved in the study
☒	☐ ChIP-seq
☒	☐ Flow cytometry
☒	☐ MRI-based neuroimaging

Antibodies

Antibodies used	Commercially available immunoassay panels from two platforms were used to quantify over 1400 proteins in the samples. Each individual protein assay relied on an antibody pair (comprised of monoclonal and/or polyclonal antibodies) directed against the protein of interest.
Validation	Validation of the immunoassay utilizing antibodies for the selected proteins in the custom assay panel is referenced in the manuscript: Qureshi, F., et al. Analytical validation of a multi-protein, serum-based assay for disease activity assessments in multiple sclerosis. Proteomics Clin Appl, e2200018 (2023)
